# The impact of neoadjuvant systemic treatment on postoperative complications in breast cancer surgery

**DOI:** 10.1007/s10549-022-06811-0

**Published:** 2022-11-20

**Authors:** R. L. Nussbaumer, N. Maggi, L. Castrezana, L. Zehnpfennig, F. D. Schwab, J. Krol, I. Oberhauser, W. P. Weber, C. Kurzeder, M. D. Haug, Elisabeth A. Kappos

**Affiliations:** 1 Department of Obstetrics and Gynecology, University Hospital of Basel, Basel, Switzerland; 2 Breast Center, University Hospital of Basel, Basel, Switzerland; 3 University of Basel, Basel, Switzerland; 4 Department of Plastic, Reconstructive, Aesthetic and Hand Surgery, University Hospital of Basel, Basel, Switzerland

**Keywords:** Breast cancer surgery, Systemic therapy, Surgery complications, Neoadjuvant

## Abstract

**Purpose:**

The aim of the study was to analyze the impact of neoadjuvant systemic treatment (NST) on postoperative complications and the beginning of adjuvant treatment.

**Methods:**

This study includes data from a prospectively maintained database including patients with breast cancer (BC) stage I–IV with or without NST undergoing breast cancer surgery between January 2010 and September 2021.

**Results:**

Out of 517 enrolled patients, 77 received NST, 440 had primary breast surgery. After NST patients underwent surgery after a meantime of 34 days (26.5–40 days). No statistical significance could be found comparing the complication grading according to the Clavien Dindo classification. The complications were most frequently rated as grade 3b. There were no complications with grade 4 or higher. When differentiating into short and long-term, the overall rate of short-term complications was 20.3% with no significant difference between the two groups (20.8% vs. 20.2%). Regarding long-term complications, there was more impairment of shoulder mobility (26.0% vs. 9.5%, *p* ≤ 0.001) and chronic pain (42.9% vs. 28.6%, *p* ≤ 0.016) for patients with NST. The beginning of the administration of the adjuvant treatment was comparable in both groups (46.3 days vs. 50.5 days).

**Conclusion:**

In our cohort, complications between both groups were comparable according to Clavien Dindo. This study shows that NST has no negative impact on postoperative short-term complications and most importantly did not lead to a delay of the beginning of adjuvant treatment. Therefore, NST can be safely admitted, even when followed by extensive breast reconstruction surgery.

## Introduction

In 1970 neoadjuvant systemic treatment (NST) was introduced as a salvage treatment for patients with non-metastatic inoperable breast cancer (BC). Since then, NST has gained popularity and increasingly became an important treatment option in patients with BC.

As a result, the indication for NST nowadays extends far beyond inoperable BC. It is regularly used for downstaging large, but operable BC to reduce the extent of surgery and offer the patient the possibility of breast conserving options [1, 2]. Moreover, even in smaller tumors, NST is regarded as the standard of care, especially in patients with triple-negative breast cancer (TNBC) and Her2-positive BC. [3] In these patient populations, the response to NST has a prognostic significance and a major impact on further treatment decisions [4–9]. Despite these proven advantages of NST, there are still reservations regarding the postoperative course due to its cytotoxicity. Concerns are, that side-effects like the weakening of the immune system and restricted production of fibroblasts lead to more wound infection and wound healing problems [10]. Resulting in a delay of adjuvant treatment and consequently a worse oncological outcome.

Simultaneously surgical techniques have also developed immensely in the past decades. Originally, the focus of BC surgery was mainly on oncological safety, since disease-free survival depended on surgical treatment. With the development of additional adjuvant treatment and earlier diagnosis through screening mammographies, the rate of long-term survivors in BC patients grew rapidly. As a result, efforts regarding aesthetic results and quality of life became an essential cornerstone in the treatment of BC. Instead of a conventional mastectomy, breast-conserving oncoplastic techniques are increasingly performed and becoming the gold standard, combining oncological safety with aesthetically pleasing results [11–13].

To be able to best counsel the patient, it is important to understand the impact of NST on today’s often more complex surgical therapy and its postoperative course. Consequently, several studies have compared postoperative complications in patients with and without NST with contradictive outcomes.

This study aims to investigate the safety of NST concerning postoperative complications and the timing of adjuvant treatment across all types of breast cancer surgery. To achieve this goal, women treated with NST followed by any type of breast cancer surgery were compared to women who underwent primary surgery. To the best of our knowledge, this is the largest cohort to examine whether NST leads to an increase in postoperative complications and a delay in adjuvant treatment due to postoperative complications.

### Methods

#### Study design and patients

Our studied patient population originates from the oncoplastic surgery database of a tertiary referral center. The database is prospectively maintained and contains women with American Joint Committee on Cancer (AJCC) breast cancer stage 0–IV, who underwent either oncoplastic tumorectomy, conventional mastectomy or mastectomy with an immediate (implant based or autologous) reconstruction.

We included a consecutive series of patients from January 2010 to September 2021. A follow up of at least three months was mandatory.

Surgical techniques were divided into oncoplastic tumorectomy (including round block mammoplasty, V-Mammoplasty, B-Mammoplasty, Hemibatwing technique, and Reduction mammoplasty), conventional mastectomy, nipple/skin-sparing surgery (NSM/SSM) with immediate implant based reconstruction (including pre and sub pectoral placement) or immediate autologous reconstruction (including DIEP, TMG PAP flap and latissimus dorsi flap reconstruction) [13, 14]. Exclusion criteria were a loss of follow up or breast cancer stage 0 and a follow up of less than three months.

We compared patients with NST before surgery to patients who underwent primary surgery, acting as the control group.

The primary endpoint were were overall postoperative complications, as well as short- and long-term complications individually. Complications were classified and compared according to the Clavien Dindo classification. Short-term complications were defined as a delay of wound healing (> 21 days), skin and nipple necrosis due to ischemia, infection which required antibiotics ore more, hematoma, and seroma which lead to any further intervention such as puncture or operative revision and flap loss. Long-term complications were defined as fat necrosis (detected by palpation or imaging), fibrosis, axillary web syndrome, impairment of shoulder mobility, lymphedema, chronic pain and abdominal weakness. Secondary endpoint was the beginning of the adjuvant treatment.

#### Statistical analysis

Patient characteristics, tumor stage, surgical procedure, usage of adjuvant treatment, the beginning of adjuvant treatment, short- and long-term complications were analyzed for patients who underwent NST and patients without. Continuous parameters were summarized using mean values, standard deviation and compared using a *t* test. Categorical parameters were summarized by absolute frequencies, percentages and compared using a Fisher exact test for association. Poisson regression models were generated to identify potential predictors for the occurrence of long-term complications. NST (yes, no) was included into each model as a covariate. Additional covariates (age at baseline, diabetes status, BMI, nodal stage, and hypertension) were entered based on stepwise selection. The Incidence Rate Ratio (IRR) and corresponding 95% confidence intervals (CI) for significant parameters were entered into a table. None of the *p* values generated for the analysis were corrected for multiple testing; *p* values are therefore nominal and need to be interpreted accordingly. A *p* value < 0.05 was considered to be statistically significant.

## Results

### Patients, tumor and treatment characteristics

We enrolled 517 consecutive patients from the oncoplastic surgery database of our tertiary referral center. The demographic and clinicopathologic characteristics of our collective included in this study are presented in Table 1. 77 patients received NST. 440 patients underwent primary surgery. Due to small tumors or patient preference, 54 patients with HER2-overexpression or triple-negative breast cancers underwent primary surgery. Patients with NST were significantly younger (50.1 years vs. 62.2 years, *p* ≤ 0.001), had a larger extent of cancer and a higher nodal stage at the time of diagnosis. Hypertension was more common in the group with primary surgery (7.8% vs. 31.8%, *p* ≤ 0.001). There were no further significant differences between the two groups regarding other known risk factors for postoperative complications such as smoking status, diabetes, weight and cup size.

**Table 1:** Demographic characteristic, tumor stage, surgery procedure, time to follow up

	NST		Primary surgery		*p* value
	*n* = 77	%	*n* = 440	%	
Age, years	50.1 ± 12.3	n.a	62.2 ± 13.0	n.a	< 0.001
BMI, kg/m^2^	24.8 ± 5.3	n.a	25.4 ± 5.3	n.a	0.36
Comorbidity					
Hypertension	6	7.8	140	31.8	< 0.001
Heart disease	3	3.9	45	10.2	0.09
Lung disease	3	3.9	34	7.7	0.34
Diabetes mellitus	3	3.9	26	5.9	0.60
Smoker	16	20.8	75	17.0	0.83
Cup size					
A	3	3.9	28	6.4	0.35
B	19	24.7	81	18.4	
C	9	11.7	47	10.7	
D	1	1.3	25	5.7	
E and lager	2	2.6	12	2.7	
Unknown	43	55.8	247	56.1	
Clinical tumor category (cT)					
cT1	20	26.0	260	59.1	< 0.001
cT2	44	57.1	146	33.2	
cT3	9	11.7	29	6.6	
cT4	4	5.2	5	1.1	
Clinical nodal category (cN0)					
cN0	34	44.2	369	83.9	< 0.001
cN1	36	46.8	61	13.9	
cN2	2	2.6	5	1.1	
cN3	5	6.5	4	0.9	
Unknown	0	0	1	0.2	
Receptor status					
HR−/HER2−	22	28.6	17	3.9	
HR−/HER2+	8	10.4	10	2.3	
HR+/Her2+	14	18.5	27	6.1	
HR+/Her2−	15	19.5	357	81.1	
Unknown	18	23.4	29	6.6	0.000
Pathological tumor category (ypT/pT)					
ypT0	33	42.9	n.a	n.a	< 0.001
ypTis/pTis	7	9.1	8	1.8	
ypT1/pT1	30	39.0	261	59.3	
ypT2/pT2	7	9.1	138	31.4	
ypT3/pT3	0	0.0	30	6.8	
ypT4/pT4	0	0.0	1	0.2	
Unknown	0	0.0	1	0.2	
Pathological tumor category (ypN/pN)					
ypN0/pN0	54	70.1	280	63.6	0.42
ypN1/pN1	16	20.8	93	21.1	
ypN2/pN2	3	3.9	23	5.2	
ypN3/pN3	1	1.3	25	5.7	
Unknown	3	3.9	19	4.3	
Interval NST—surgery					
Interval between last dose of neoadjuvant chemotherapy and surgery, days	34 ± 14.5	n.a	n.a	n.a	n.a
Surgery procedure					
Oncoplastic surgery	45	58.4	301	68.4	0.009
Conventional mastectomy	7	9.1	64	14.5	
NSM/SSM with implant	6	7.8	28	6.4	
NSM/SSM with autologous reconstruction	19	24.7	47	10.7	
Adjuvant treatment					
Chemotherapy (Monotherapy)	1	1.3	5	1.1	< 0.001
Radiotherapy (Monotherapy)	27	35.1	22	5.0	
Endocrine therapy (Monotherapy)	6	7.8	73	16.6	
Chemotherapy + Radiation	7	9.1	18	4.1	
Chemotherapy + endocrine therapy	0	0.0	16	3.6	
Radiation + endocrine therapy	25	32.5	204	46.4	
Chemotherapy + Radiation + Endocrine Therapy	2	2.6	66	15.0	
Follow up					
Follow up period, months	28.7 ± 18.8	–	38.1 ± 23.9	–	0.001

Data are given as numbers (%) or means ± standard deviation (STD)

*NST* neoadjuvant systemic treatment, *BMI* body mass index, *HR* Hormone receptor

Patients who received NST underwent surgery after a mean time of 34 days (26.5–40 days) after completion of NST.

In both groups, oncoplastic surgery was the most common surgical technique (58.4% vs. 68.4%). Regarding mastectomy, immediate reconstruction was the preferred method in both groups.

The mean follow up time was over two years in both groups, although there was a significant difference with a follow up of 28.7 months in the NST group compared to 38.1 months in the control group (Table 1).

### Overall complications

No statistical significance could be found comparing the overall complication, when grading them according to the Clavien Dindo classification (Table 2). The complications were most frequently rated as grade 3b, without any with grade 4 or higher.

**Table 2:** Clavien Dindo classification

	NST	Primary surgery	*p* value
	*n* = 77 (%)	*n* = 440 (%)	
Grade 1	5 (6.5)	28 (6.4)	0.963
Grade 2	0 (0.0)	5 (1.1)	
Grade 3a	2 (2.6)	13 (3.0)	
Grade 3b	9 (11.7)	42 (.5)	
No complication	61 (79.2)	350 (79.5)	
Unknown	0 (0.0)	2 (0.5)	

Data are given as numbers (%)

*NST* neoadjuvant systemic treatment

### Short-term complications

The overall rate of short-term complications was comparable in both groups (20.8% vs. 20.2%, *p* = 0.879). There was no significant difference in delayed wound healing (10.4% vs. 8.0%, *p* = 0.501), infection (5.2% vs. 6.6%, *p* = 0.803), hematoma (5.2% vs. 4.3%, *p* = 0.763), seroma (11.7% vs. 8.6%, *p* = 0.391), flap loss (1.3% vs. 0.7%, *p* = 0.476) and nipple necrosis (1.3% vs. 0.7%, *p* = 0.476). We could find a trend toward more skin necrosis in the NST group (7.8% vs. 3.2%, *p* = 0.099), this did though not reach statistical significance (Table 3).

**Table 3:** Postoperative short-term complications

	NST	Primary surgery	*p* value
	*n* = 77 (%)	*n* = 440 (%)	
Overall short-term complications	16 (20.8)	89 (20.2)	0.879
Delayed wound healing	8 (10.4)	35 (8.0)	0.501
Infection	4 (5.2)	29 (6.6)	0.803
Hematoma	4 (5.2)	19 (4.3)	0.763
Seroma	9 (11.7)	38 (8.6)	0.391
Skin necrosis	6 (7.8)	14 (3.2)	0.099
Nipple necrosis	1 (1.3)	3 (0.7)	0.476
Flap loss	1 (1.3)	3 (0.7)	0.476

Data are given as numbers (%), *NST* neoadjuvant systemic treatment, delayed wound healing: > 21 days, infection: treatment with antibiotics, hematoma/seroma: required further intervention

### Long-term complications

There was no difference for most long-term complications including fat necrosis (6.5% vs. 4.5%, *p* = 0.400), fibrosis (3.9% vs. 1.6%, *p* = 0.175), axillary web syndrome (1.3% vs. 2.0%, *p* = 1.000), lymphedema (5.2% vs. 12.0%, *p* = 0.112) and abdominal weakness. But patients in the NST group had a significant higher rate of shoulder mobility impairment (26.0% vs. 9.5%, *p* =  < 0.001) and chronic pain (42.9% vs. 28.6%, *p* = 0.016) (Table 4). Accordingly, our multivariate analysis revealed that receiving NST (incidence rate ratio (IRR, 2.8; *p* ≤ 0.001), as well as younger age (per 5 years; IRR, 1.12; *p* = 0.025), was associated with impairment of shoulder mobility (Table 5). Both factors showed as well an influence on chronic pain (NST IRR, 2.05; *p* ≤ 0.001; age OR 1.1; *p* = 0.002) (Table 5).

**Table 4:** Postoperative long-term complications

	NST	Primary surgery	*p* value
	*n* = 77 (%)	*n* = 440 (%)	
Overall long-term complications	48 (62.3)	205 (46.6)	0.013
Fat necrosis	5 (6.5)	20 (4.5)	0.400
Fibrosis	3 (3.9)	7 (1.6)	0.175
Axillary web syndrome	1 (1.3)	9 (2.0)	1.000
Lymphedema	4 (5.2)	53 (12.0)	0.112
Impairment of shoulder mobility	20 (26.0)	42 (9.5)	< 0.001
Chronic pain	33 (42.9)	126 (28.6)	0.016
Abdominal hernia	0 (0.0)	3 (0.7)	1.000
Atrophy	0 (0.0)	1 (0.2)	1.000
Relaxation	1 (1.3)	0 (0.0)	0.149

Data are given as numbers (%); *NST* neoadjuvant systemic treatment, fat necrosis: palpable or detected by sonography

**Table 5:** Multivariate analysis for long-term complications

	IRR	95% CI	*p* value
Chronic pain			
NST			
No	Ref	1.40–2.99	
Yes	2.05	0.86–0.97	< 0.001
Age (years)			
Per 5 years increase	0.91		0.002
Impairment of shoulder mobility			
NST			
No	Ref	1.58–4.95	< 0.001
Yes	2.80	0.81–0.99	0.025
Age (years)			
Per 5 years increase	0.89		

*IRR* incidence rate ratio, *NST* neoadjuvant systemic treatment; NST (yes, no) was included in the model as covariate. Additional covariates, including age at baseline, nodal Stage (< pN1, pN1, > pN1), BMI (< 18.5, 18.5 – < 25, 25 – < 30, ≥  30), Diabetes (Yes, No), Hypertension (Yes, No), were entered into the model based on the stepwise selection. Nine observations were deleted due to missing values

### Adjuvant treatment

Table 6 compares the time between surgery and the beginning of the adjuvant treatment overall. We could not find a statistically significant difference between NST and the control group (46.3 days vs. 50.5 days, *p* = 0.300).

**Table 6:** Timing of adjuvant treatment

	NST		Primary surgery		*p* value
	*n*	Days (mean) ± STD	*n*	Days (mean) ± STD	
Time to adjuvant treatment overall	54	46.3 ± 16.3	296	50.5 ± 28.6	0.300
Time to adjuvant chemotherapy	9	47.1 ± 35.1	98	43.8 ± 25.7	0.718

*NST* neoadjuvant systemic treatment

## Discussion

In the past decade, NST has become increasingly important in the treatment of BC. With NST, mastectomy can often be omitted in favor of breast-conserving therapy [16–18]. Resulting in an often smaller operation and a shorter operating time with a good aesthetic result. In cases, where a mastectomy is still necessary regarding oncological safety, immediate reconstruction became more popular. Compared to conventional mastectomy, immediate reconstruction leads to a better aesthetic result with an improved quality of life [19, 20]. However especially in cases with complex and extensive breast surgery, there are still reservations about the neoadjuvant setting. Chemotherapy targets rapidly dividing cells, which affects not only cancer cells. Myelosuppression is a well-known side effect, which leads to a reduction in leukocytes. It causes a weakening of the patient’s immune system, making her more susceptible to infections. In addition, fibroblast production and collagenous synthesis, both essential in the wound healing process, are restricted. This might have a negative influence on the postoperative course by leading to delayed wound healing [10, 12]. Further, due to endothelial dysfunction, platelets are increasingly activated, which raises the risk of thrombosis [21, 22]. This is known to cause flap loss after autologous reconstruction.

This study aimed to examine the impact of NST on the postoperative course, regarding postoperative complications and its effect on the beginning of adjuvant treatment, including all types of breast cancer surgery.

### Overall, short-term and long-term complications

Two important findings have emerged from the current study. Firstly, we could not find any negative impact between administered NST and overall complications according to the Clavien Dindo classification. Secondly, there was no difference in postoperative short-term complications. These results are especially relevant to informing therapeutic choices, as in consequence, there was no delay in the adjuvant treatment for patients with NST. There was a slight trend towards more skin necrosis in the NST group. Regarding the small numbers of only 6 skin necrosis in the NST group compared to 14 in the group undergoing primary surgery (7.8% vs. 3.2%, *p* = 0.099) out of 517 included patients, those results are only of marginal evidence.

Concerning long-term complications, we could find significantly more often chronic pain and impairment of shoulder mobility in the NST group. Since patients with a higher nodal stage are more likely to receive NST and more extensive axillary surgery, we expected that their nodal status might be associated with increased chronic pain and impairment of shoulder mobility. Interestingly, additional multivariate analysis to answer this specific question revealed that younger age and NST were associated with both long-term complications. This should be taken into account for perioperative patient management such as physiotherapy and pain management. Furthermore, both chronic pain and impairment of shoulder mobility did however not lead to a delay in adjuvant treatment [11, 23, 24].

Our results concerning the postoperative complication rate following NST confirm the conclusion of a recently published systematic review and meta-analysis by Lorentzen et al.[25] They searched the literature for studies assessing the impact of NST on postoperative complications, comparing a group of patients receiving NST to a control group. According to Lorentzen et al. NST is not associated with increased postoperative complications. The studies included in this meta-analysis though all investigated the complication rates after NST regarding a specific surgical technique. In the current study, we specifically aimed to include patients across all breast surgical procedures. With nearly no exclusion criteria, it reflects the patient population of most of the breast surgery departments around the world in a pragmatic, real life setting.

### Interval between NST and surgery

Regarding the overall survival and recurrence rate, surgery following NST should be performed as soon as possible to ensure the best possible outcome. Meanwhile, a too short interval may lead to an increased risk of postoperative complications. In our trial the mean time between completion of NST and surgery was 34 days (26.5–40 days). This adequate interval is supported by current data recommending an interval of 4–8 weeks [26].

### Interval between surgery and adjuvant treatment

For an optimal oncological outcome, the timely start of adjuvant treatment is essential [27–29]. A meta-analysis from Biagi et al. demonstrated a 6% increased risk for death every 4 weeks of delay of the adjuvant chemotherapy [30]. Especially for patients with hormone receptor-negative cancer initiation of adjuvant treatment during the first three postoperative weeks seems to be crucial to offer the patients the best possible outcome [31, 32]. In hormone receptor-positive cancer, an interval of no more than 8–12 weeks should be observed [32]. Regarding adjuvant radiotherapy, the interval should also be kept as short as possible within the first twelve weeks postoperatively [27, 29, 33, 34].

Due to severe postoperative complications, initiation of adjuvant chemo- or radiotherapy may be delayed. In our study, we were able to show, that across all types of oncoplastic procedures, patients with NST started adjuvant treatment at the same time as patients undergoing primary surgery. These results confirm the data of a few smaller retrospective reviews indicating, that NST has in fact no negative impact on the administration of adjuvant treatment [35, 36].

Due to severe postoperative complications, initiation of adjuvant chemo- or radiotherapy may be delayed. In our study, we were able to show, that across all types of oncoplastic procedures, patients with NST started adjuvant treatment at the same time as patients undergoing primary surgery. These results confirm the data of a few smaller retrospective reviews indicating, that NST has in fact no negative impact on the administration of adjuvant treatment [30, 31].

### Strengths and limitations

The most important limitation to our study is based on its retrospective design, resulting in two very unevenly sized groups. Whereas most of the baseline characteristics were comparable, including well-known risk factors for postoperative complications such as obesity, cup size, smoking and diabetes,- there was a significant difference in age, initial TNM-classification and applied surgical procedure. The NST group was significantly younger, had a higher TNM-classification at initial diagnosis and underwent more often a mastectomy with reconstruction. This selection bias can be explained by tumor biology, patients characteristics and surgeons choice. Aggressive tumor biologies (HER-2 positive BC, TNBC) are more likely in younger patients and require NST. Meanwhile younger patients seem to elect more often to undergo reconstructive breast surgery, aiming for the best possible aesthetic result, while surgeons tend to recommend the least invasive surgery to older patients to decrease the risk of complications even further [32].

There are several strengths to our study. We used only marginal exclusion criteria, which makes this study unique, depicting a real world setting of a tertiary breast cancer referral center. Previous studies investigating the effect of NST on the postoperative course, always focused on one specific surgical technique only, limiting the generalizability of their results. Our study examined the impact of NST across all patients undergoing BC surgery. Therefore, our patient population reflects the actual real world data within today’s treatment guidelines. Consequently, our results might be transferred more accurately to any other breast center. Another strength of the study is the size of our patient cohort. To our knowledge, it is to date the biggest patient cohort, looking at the effects of NST as a potential adjuvant treatment delay.

## Conclusion

In our cohort of 517 patients, NST did not lead to an increased rate of postoperative short-term complications nor a delay in adjuvant treatment regardless of surgical procedure. Therefore, NST can be safely administered if indicated, regardless of the extend of surgery.

## Data Availability

The datasets generated during and/or analyzed during the current study are available from the corresponding author on reasonable request.
